# Supplementary material for: MetaRibo-Seq measures translation in microbiomes
Source: Nat Commun. 2020 Jun 29;11:3268. doi: 10.1038/s41467-020-17081-z (PMC7324362; doi:10.1038/s41467-020-17081-z)
Supplement: Supplementary file 10 — Supplementary Data 7 [file 41467_2020_17081_MOESM10_ESM.zip › File2/Confidence_High_Taxonomy/132841_out.krona.html]

Javascript must be enabled to view this page.

members
magnitude
magnitudeUnassigned
count
unassigned
taxon
rank

132841\_out

70

superkingdom
2
70

1239
70
phylum

186801
70
class

order
10
70
186802

SRS015217\_contig\_number\_contig-100\_2206.183810SRS017433\_contig\_number\_contig-100\_33715.33715SRS019445\_contig\_number\_contig-100\_3143.203498SRS049959\_contig\_number\_contig-100\_2116.270602SRS052027\_contig\_number\_contig-100\_13474.48275SRS064276\_contig\_number\_22039SRS076804\_contig\_number\_contig-100\_31651.31652SRS104311\_contig\_number\_17108SRS142890\_contig\_number\_contig-100\_5745.249586SRS971275\_contig\_number\_contig-100\_12910.83835

20
186803
family

1
189330
2

SRS050752\_contig\_number\_11002
genus

39486
1
species

SRS147022\_contig\_number\_15102

genus

SRS049900\_contig\_number\_contig-100\_3840.106008SRS1041031\_contig\_number\_contig-100\_2510.152107SRS104636\_contig\_number\_contig-100\_19912.19913SRS143342\_contig\_number\_contig-100\_24155.65667
4
572511
18

1
418240
species

SRS020233\_contig\_number\_contig-100\_532.342879

1226324
2

SRS1054716\_contig\_number\_contig-100\_9236.75794SRS143466\_contig\_number\_contig-100\_2332.157356
species

4
1737424

SRS014979\_contig\_number\_contig-100\_2895.182207SRS016495\_contig\_number\_contig-100\_1744.119853SRS050941\_contig\_number\_7118SRS148511\_contig\_number\_15239
species

765821
7

SRS018351\_contig\_number\_19552SRS049773\_contig\_number\_26353SRS076876\_contig\_number\_1148SRS077502\_contig\_number\_9559SRS1041118\_contig\_number\_4338SRS147977\_contig\_number\_contig-100\_5069.45125SRS148253\_contig\_number\_11440
species

family
3
31979

genus
1485
3

3
59620
species

SRS019068\_contig\_number\_contig-100\_689.661929SRS023526\_contig\_number\_contig-100\_2376.232029SRS893378\_contig\_number\_contig-100\_3229.45546

family
37
541000

genus

SRS012273\_contig\_number\_48880SRS017521\_contig\_number\_contig-100\_1726.264641SRS020394\_contig\_number\_contig-100\_5314.5315SRS023971\_contig\_number\_contig-100\_9900.9900SRS063127\_contig\_number\_contig-100\_3251.35299SRS077454\_contig\_number\_contig-100\_9261.82694SRS100021\_contig\_number\_contig-100\_9570.49597SRS1041136\_contig\_number\_contig-100\_4550.115546SRS893252\_contig\_number\_5515
37
1263
9

2
2293228
species

SRS015431\_contig\_number\_62821SRS148091\_contig\_number\_5932

species

SRS016267\_contig\_number\_contig-100\_1018.38289SRS042284\_contig\_number\_contig-100\_1711.49685SRS043411\_contig\_number\_14616SRS053214\_contig\_number\_contig-100\_1285.132040SRS062701\_contig\_number\_contig-100\_1512.68885SRS075398\_contig\_number\_contig-100\_1394.1395SRS097889\_contig\_number\_contig-100\_908.228562SRS147377\_contig\_number\_contig-100\_2188.43641SRS148159\_contig\_number\_contig-100\_3528.334302SRS893270\_contig\_number\_20138
2293151
10

657323
8

SRS015663\_contig\_number\_contig-100\_2336.198590SRS023914\_contig\_number\_20813SRS050422\_contig\_number\_27994SRS051882\_contig\_number\_contig-100\_1579.138115SRS063518\_contig\_number\_contig-100\_8743.129361SRS077552\_contig\_number\_9632SRS144183\_contig\_number\_contig-100\_3305.193097SRS147139\_contig\_number\_contig-100\_16037.166984
species

species

SRS143991\_contig\_number\_19584
1
1262968

2293188
4
species

SRS063040\_contig\_number\_contig-100\_2625.247328SRS1041033\_contig\_number\_29382SRS1041091\_contig\_number\_17560SRS149181\_contig\_number\_contig-100\_1935.170404

1
2293224

SRS012969\_contig\_number\_8779
species

2293190
2
species

SRS147346\_contig\_number\_contig-100\_4533.312904SRS148721\_contig\_number\_55621
